# Echocardiographic Parameters to Predict Malignant Events in Arrhythmic Mitral Valve Prolapse Population

**DOI:** 10.3390/jcm12031232

**Published:** 2023-02-03

**Authors:** Alessandro Vairo, Paolo Desalvo, Andrea Rinaudo, Francesco Piroli, Anna Tribuzio, Andrea Ballatore, Gianluca Marcelli, Lorenzo Pistelli, Veronica Dusi, Nicolò Montali, Gianluca Alunni, Cristina Barbero, Stefano Salizzoni, Marco Pocar, Mauro Rinaldi, Fiorenzo Gaita, Gaetano Maria De Ferrari, Carla Giustetto

**Affiliations:** 1 Division of Cardiology, Cardiovascular and Thoracic Department, Città della Salute e della ScienzaHospital, 10126 Turin, Italy; 2 Division of Cardiac Surgery, Department of Surgical Sciences, Città della Salute e della Scienza di Torino, University of Turin, 10126 Turin, Italy

**Keywords:** mitral valve prolapse, echocardiography, ventricular arrhythmia, sudden cardiac death

## Abstract

Bileaflet Mitral Valve Prolapse (bMVP) has been linked to major arrhythmic events and sudden cardiac death (SCD). Consistent predictors in this field are still lacking. Echocardiography is the best tool for the analysis of the prolapse and its impact on the ventricular mechanics. The aim of this study was to find new echocardiographic predictors of malignant events within an arrhythmic MVP population. We evaluated 22 patients with arrhythmic bMVP with a transthoracic echocardiogram focused on mitral valve anatomy and ventricular contraction. Six of them had major arrhythmic events that required ICD implantation (ICD-MVP group), while sixteen presented with a high arrhythmic burden without major events (A-MVP group). The best predictors of malignant events were the Anterior Mitral Leaflet (AML) greater length and greater Mechanical Dispersion (MD) of basal and mid-ventricular segments, while other significant predictors were the larger mitral valve annulus (MVA) indexed area, lower MVA anteroposterior diameter/AML length ratio, higher inferolateral basal segment S3 velocity.

## 1. Introduction

**Figure 1:**
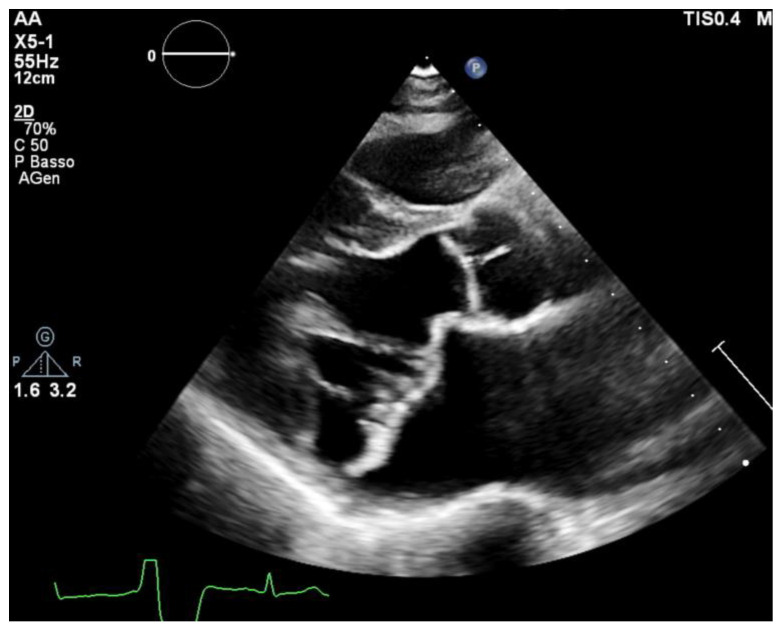
A parasternal long axis (PLAX) view of MVP in end-systole from a patient enrolled in the aMVP group. Note the prolapse of both leaflets above the mitral annulus plane.

Mitral Valve Prolapse (MVP) is a pathology that affects up to 3% of the general population, and one third of this group has a form of prolapse involving both leaflets (bileaflet MVP or bMVP) [1,2]. Echocardiography is considered the gold standard imaging modality for MVP diagnosis. MVP is defined as a displacement of the mitral leaflets more than 2 mm into the left atrium during systole (Figure 1). Histologically, it can vary within a wide spectrum of types from the classical form (in which the leaflets have a maximal thickness of at least 5 mm during diastole) to the non-classical form with thinner leaflets due to a fibroelastic deficiency [3,4]. MVP prognosis can vary largely depending on multiple variables, such as the degree of mitral regurgitation (MR), left ventricular ejection fraction (EF), ventricular ectopics, atrial diameters and patient’s age [5,6]. Ever since its first description, MVP had been linked with ventricular arrhythmias (VAs) and sudden cardiac death (SCD) [6,7,8]. More recent observational studies have highlighted a concrete risk of SCD and sustained ventricular tachyarrhythmias, with an estimated annual risk ranging from 0.2% to 1.9% [9,10].

Therefore, early identification of the patients at risk of MVP-related SCD is crucial to prevent potentially lethal adverse events. Despite being still poorly understood, the substrate for arrhythmias seems to be the cardiac fibrosis induced by abnormal tension on the papillary muscle and on the basal inferolateral myocardium. Both MRI and histologic studies have confirmed the presence of a scar in these areas [10]. Current theories about the VA triggers are focused on the traction on the papillary muscle and on the basal inferolateral wall [11] and its effect on the Purkinje fibers that are localized near these sections of the myocardium. Traction on the papillary muscle can prolong the ventricular refractory period, which may induce or sustain papillary muscle arrythmias. In addition, it has been observed that some Purkinje fibers can arborize in the area adjacent to the papillary muscle and in the inferolateral myocardium [12]. These fibers may possibly act as a trigger for VAs.

Many risk factors have been proposed as predictors of adverse events in patients with MVP, but predictors of major VAs in MVP remain poorly established despite the growing interest in this topic.

## 2. Materials and Methods

This is a monocentric retrospective study comparing two groups of patients with arrhythmic MVPs. Patients were followed at the University Hospital of Turin (Città della Salute e della Scienza) over the last 24 years. After years of follow-up, most of these patients were included in a Register of Arrhythmic Mitral Valve Prolapse, which was created in 2020 and approved by the Institutional Ethics Committee of A.O.U. Città della salute e della scienza di Torino—A.O. Ordine Mauriziano di Torino—A.S.L. Città di Torino—protocol code 0036661—date of approval was 10 April 2020. The register includes 72 patients, and we selected those with a previous major arrhythmic event and those with high arrhythmic burden and high-risk features to carry out the present investigation. Inclusion criteria were the presence of bileaflet MVP and a high burden of ventricular arrhythmias, which had been documented at a previous ECG Holter (defined as a ventricular burden of at least 1000 premature ventricular beats per day or the presence of multiple couplets, triplets, non-sustained ventricular tachycardias or sustained ventricular tachycardias) or history of previous aborted SCD.

Exclusion criteria were having previously undergone cardiac surgery, the concomitant presence of any other cardiomyopathy, intraventricular conduction defect and previous episodes of atrial fibrillation, atrial flutters or any supraventricular tachyarrhythmia that may influence the left atrial volume or mitral valve annulus size.

From the 72 patients present in the register, 17 of them were excluded due to low arrhythmic burden, 12 of them were excluded due to previous heart surgery and 5 of them were excluded because they presented with prolapse of a single leaflet.

We therefore included 38 potential patients in this study. Out of the 38 patients, 16 of them did not agree to be enrolled in the study due to logistic restraints or personal choice.

Twenty-two patients were screened with a specific echocardiographic protocol in the period from March 2021 to October 2021.

Patients were divided into two groups based on history of previous major arrhythmic events leading to an implantable cardioverter defibrillator (ICD) implantation in secondary prevention (ICD-MVP group, 6 patients: 5 of them had an aborted SCD, 1 of them had a syncopal sustained ventricular tachycardia)- or the presence of VAs only (A-MVP group, 16 patients).

The study was conducted according to the Declaration of Helsinki, and all of the patients provided written informed consent for inclusion in the present study. The study protocol was reviewed by local Ethics Committee.

**Figure 2:**
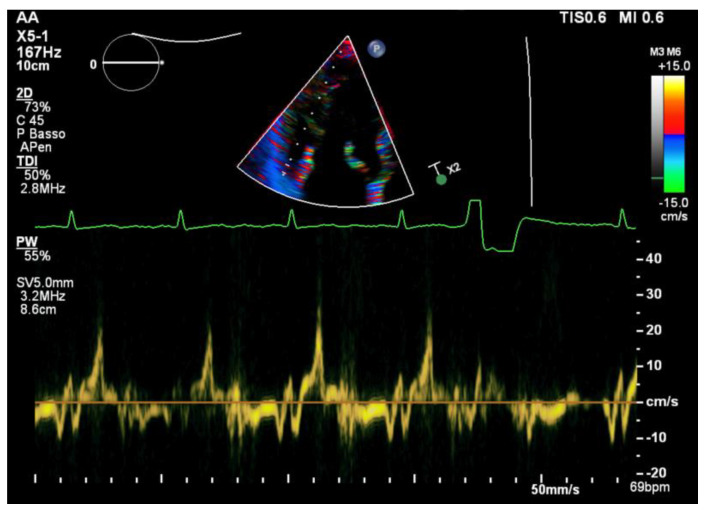
A pulsed wave tissue Doppler signal on the basal inferolateral wall from the same patient of Figure 1 (aMVP group). Note the spiked high velocity signal (Pickelhaube sign).

All of the patients underwent transthoracic echocardiography with ECG tracing. Echocardiography was performed using a Philips EpiQ CvX 7C echocardiograph with a Philips X5-1 3D probe. Every patient was screened with basal echocardiography, with careful morphological evaluation of the mitral valve (MV) and grading of the mitral regurgitation (MR) [13], along with a specific protocol to investigate tissue Doppler imaging of the mitral annulus, 3D imaging of the mitral valve and 3D full volume reconstruction of the ventricle. Bileaflet MVP was defined as the prolapse of both leaflets into the left atrium above the mitral annulus by at least 2 mm during the end-systole period, which is visualized in parasternal long axis view (PLAX), as shown in Figure 1 [14]. Mitral valve leaflets’ thickness and length were measured with 2D imaging in PLAX in meso-diastole, whereas the maximum height of the prolapse was measured during the end-systole period. Leaflet thickness was measured in diastole at the thickest point of the leaflet. Leaflets were defined myxomatous based on their morphology and thickness (greater than 5 mm). Antero-posterior and inter-commissural diameters of the mitral valve annulus (MVA) were measured in 2D in PLAX and in A2C views, respectively, both during the diastole and end-systole periods to investigate the presence of paradoxical systolic annular expansion [15]. Antero-posterior and inter-commissural diameters were also measured with 3D imaging, along with the MVA circumference and area. The anterior mitral leaflet (AML) length/AP diameter ratio was also calculated [13]. All of the anatomical parameters were tested at the baseline and after an adjustment for Body Surface Area (BSA). The Tissue Doppler Pulsed Wave (TDI-PW) was measured with an effort to optimize the ultrasound beam alignment with the longitudinal motion of the MVA on both the basal left ventricular segments displayed in the apical 4 chambers view (A4C), apical 2 chambers view (A2C) and apical 3 chambers view (A3C), thus providing 6 tracings for each patient. In this article, we refer to the “spiked systolic high-velocity signal” described by Muthukumar et al. as S3 [16], example shown in Figure 2. Absolute values of S1, S2 and S3 waves were measured. Mechanical dispersion was measured using the TDI, speckle tracking and Electro-Mechanical Window (EMW). The time from the onset of the R wave to the onset of the S1 wave, the end of S2/S3 wave, and the peak of S3 wave were measured using the simultaneous ECG monitoring to assess the presence of dyssynchrony by calculating the standard deviation of each time in all of the six TDI pulsed wave Doppler tracks within the same patient [17]. The speckle tracking (STE) analysis was performed on the workstation using the software CMQ 10 for Q-LAB, and global longitudinal strain (GLS) was calculated using the A4C, A2C and A3C views with a good display of the endocardial border. The endocardial border was outlined by a single operator at the end-diastolic phase of the cardiac cycle. Minor adjustments were made to optimize tracking throughout the cardiac cycle. Mechanical dispersion (MD) was calculated as the standard deviation (SD) of the times measured from the onset of R on the ECG to the peak strain (TPS) in each of the 18 segments of the left ventricle, and sub-analyses were performed separately considering the basal segments alone, the mid-ventricular segments alone, the apical segments alone and basal segments combined with middle segments as well. The transversal shift measured by CMQ 10 using Q-LAB software was analyzed as well in order to quantify the transversal motion of the ventricular walls throughout the cardiac cycle. Additionally, the GLS pattern was analyzed to research a specific double peak pattern that is suggestive of arrhythmic events and dyssynchrony.

Finally, the Electro-Mechanical Window (EMW) was calculated as the difference between the time interval from the QRS onset to aortic valve closure, as measured on the continuous-wave Doppler image in the A3C view and the QT interval during the same cardiac cycle.

### Statistical Analysis

The Statistical Package for Social Sciences (SPSS Inc., Chicago, IL, USA) was used for the statistical analyses. Continuous variables’ distribution was assessed for normality using Shapiro–Wilk test.

The comparison between the two groups (ICD-MVP vs. A-MVP) was performed using Student’s t-Test for continuous normally distributed variables, and Mann–Whitney U Test for continuous non-normally distributed variables. The categorical variables’ differences between each group were assessed with Χ2 test. Categorical variables are expressed as percentages. *p*-values < 0.05 are considered to be statistically significant.

A receiver operating characteristic (ROC) curve was created for each variable presenting a significant difference between the two groups to analyze their ability to predict major arrhythmic events. An area under the curve (AUC) of 0.6 or below was considered to be poor, while an AUC of 0.8 was considered to be good and was taken as the minimum cut off accepted for our study. The ROC curves were compared with Cohen’s Kappa index to establish inter-rater reliability. Youden’s index was used to identify the parameter combining best specificity and sensitivity for each variable.

## 3. Results

Population characteristics are summarized in Table 1.

In the ICD-MVP group, the first major arrhythmic event happened at a mean age of 36.7 ± 13.6 years. After the first episode, genetic analyses were performed in all six patients to identify gene mutations compatible with long QT syndromes, Brugada syndrome and polymorphic catecholaminergic ventricular tachycardia. Two patients had a negative screening, one patient had a mutation in the Calcium Channel gene CACNA1C, two patients in Sodium Channel genes (SCN9A and SCN5A), while the last one in a Potassium Channel gene (KCNE1). All these variants were of uncertain significance (VUS). In the A-MVP group, instead, only one patient underwent genetic analyses due to the finding of a long QT at the basal ECG, which resulted positive for a mutation in the KCNQ1 gene, pathogenetic for LQTS1.

As shown in Table 2, the anatomical echocardiographic parameters such as anterior mitral leaflet (AML) length (ICD-MVP: 27.4 ± 4.5 mm, A-MVP: 22.6 ± 2.9 mm; *p* = 0.03), MVA indexed area (ICD-MVP: 7.0 ± 1.3 cm^2^/m^2^, A-MVP: 5.7 ± 1.3 cm^2^/m^2^; *p* = 0.02) and AML length/AP diameter ratio (ICD-MVP: 1.29 ± 0.3, A-MVP: 1.47 ± 0.2; *p* = 0.049) were significantly different between the two groups. In contrast, the maximum height of the prolapse, AML thickness, posterior mitral leaflet (PML) length, PML thickness, antero-posterior (AP) and inter-commissural (IC) diameters in both the end-diastole and end-systole periods and the MV indexed annulus circumference, despite it being increased in the ICD-MVP group, did not reach statistical significance.

Mitral Annular Disjunction (MAD) was identified in four patients of the ICD-MVP group, and in nine patients in the aMVP group. Neither its prevalence, nor its entity were significantly different in the comparison between the two groups.

The results of the functional parameters analysis, which are listed in Table 3, shows that the grading of mitral regurgitation did not differ between the two groups, and there is a prevalence of mild-to-moderate mitral regurgitation.

In the speckle tracking analyses, the longitudinal strain of single segments was similar between the two groups. The transversal shift, which was measured with speckle tracking, did not result significantly different between the cases and the controls. The strain pattern analyses highlighted the presence of a double peak pattern in both populations, both for longitudinal and transversal motions, but the incidence was similar between the two groups.

Mechanical dispersion calculated with speckle tracking analysis did not differ significantly between the two groups when it was measured globally, including all of the 18 segments of the left ventricle (ICD-MVP: 67.4 ± 23 ms, A-MVP: 70.0 ± 33 ms; *p* = 0.80).

When it was analyzed by single segments (basal, middle and apical), the mechanical dispersion of the apical segments did not differ between the two groups, while the basal and middle segments showed each an increased mechanical dispersion in the ICD-MVP group that did not reach statistical significance when it was analyzed alone (basal or middle), but it was statistically significant when it was analyzed in a combined way (ICD-MVP: 117 ± 31 ms, A-MVP: 76 ± 48 ms; *p* = 0.03).

Instead, the mechanical dispersion calculated for the combination of apical and basal segments or for the combination of middle and apical segments did not differ between the two populations.

The measurement of mechanical dispersion through the TDI did not highlight any significant difference between the two groups’ times, nor did the measurement of the Electro-Mechanical Window.

In the TDI analyses, the S3 velocity of the inferolateral basal segment was significantly increased in the ICD-MVP population (ICD-MVP: 28.2 ± 11.7; A-MVP: 16.5 ± 11.1; *p* = 0.02).

The remaining S1, S2 and S3 measurements did not differ significantly in any segment between the two groups.

Annular diameters paradoxical expansion was measured as the ratio between the end-diastolic diameter EDD and the end-systolic diameter (ESD). Both of the groups presented paradoxical systolic expansion, but no differences were found between the two groups.

In summary, out of all of the echocardiographic parameters analyzed, those that were significantly different in the ICD-MVP group were AML length, AML length/AP diameter ratio, MV area (both indexed or in absolute values), the mechanical dispersion of the basal and middle segments combined calculated with speckle tracking analyses and the S3 velocity of the inferolateral basal segment.

**Figure 3:**
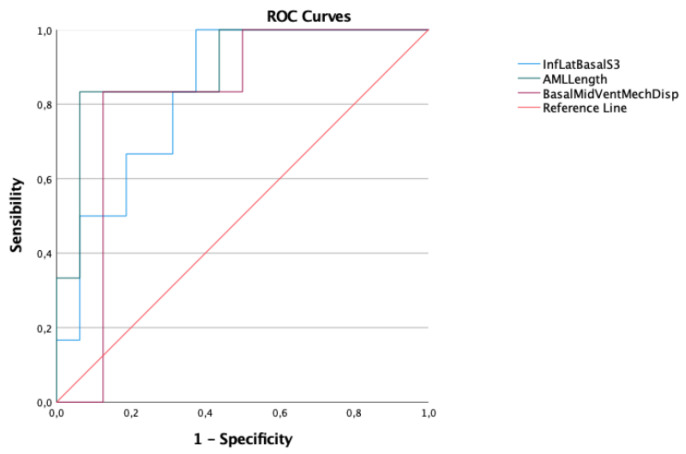
AML length, basal/mid-ventricular MD and inferolateral basal S3 ROC curves.

The ROC curves were calculated for each of these parameters. Those with AUC > 0.80 are shown in Figure 3.

In Table 4 the AUC of each ROC curve is displayed. Two variables were excluded due to an AUC value smaller than 0.80.

AML length was the variable that stood out as the best predictor, with the highest AUC. Kappa Cohen Test was used to compare it the other two variables, showing a good correlation with the Basal/Mid-Ventricular mechanical dispersion (0.60) and a moderate correlation with inferolateral basal segment S3 velocity (0.56).

**Figure 4:**
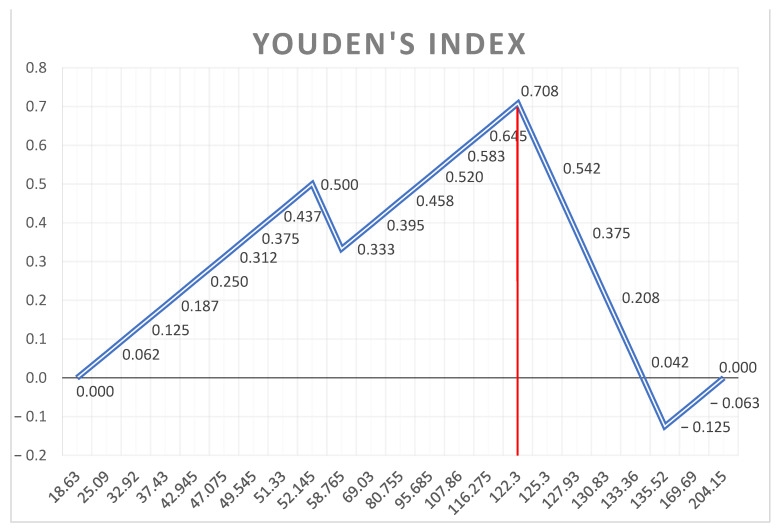
Youden’s Index of the ROC curve of the Basal and Mid-Ventricular segments mechanical dispersion.

**Figure 5:**
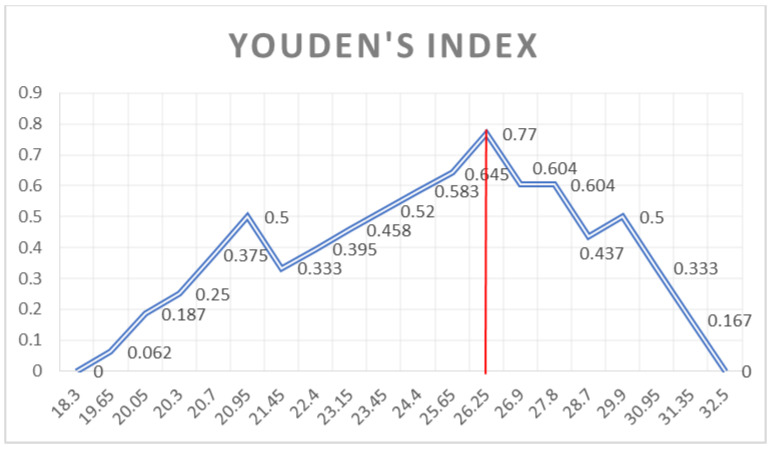
Youden’s index of the AML length ROC curve.

**Figure 6:**
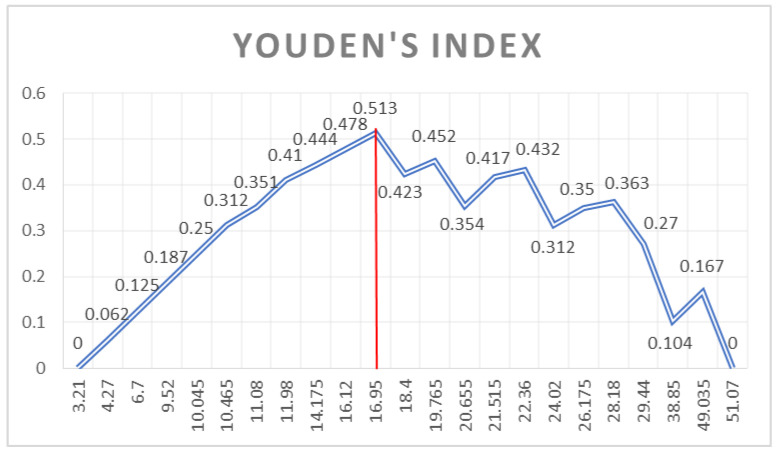
Youden’s Index of the inferolateral basal segment S3 velocity ROC Curve.

Youden’s Index (shown in Figure 4, Figure 5 and Figure 6) was used to identify which cut-off value would combine the best sensitivity and specificity for each variable that was kept as a valid predictor, in accordance with the pre-specified AUC cut-off value of 0.80.

**Figure 7:**
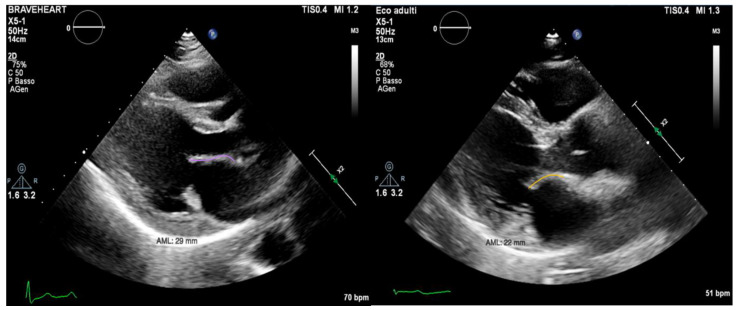
On the left, PLAX (parasternal long axis) view of a patient from the ICD-MVP group, with a longer AML (anterior mitral leaflet) (29 mm). On the right, PLAX view of a patient from the A-MVP group, with a shorter AML (22 mm).

**Figure 8:**
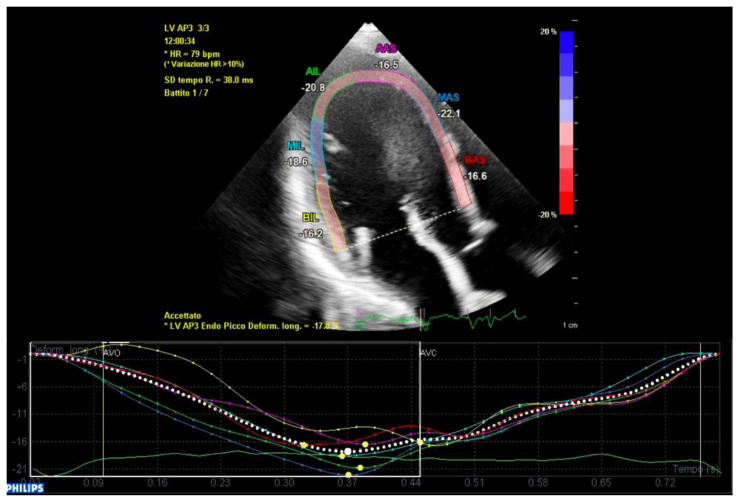
Mechanical dispersion in a patient from the ICD-MVP group. The segments that present greater peak systolic shortening time dispersion are the anterior-septal basal segment (in red) and the inferolateral basal segment (in yellow). Apical segments present a synchronous peak systolic shortening.

**Figure 9:**
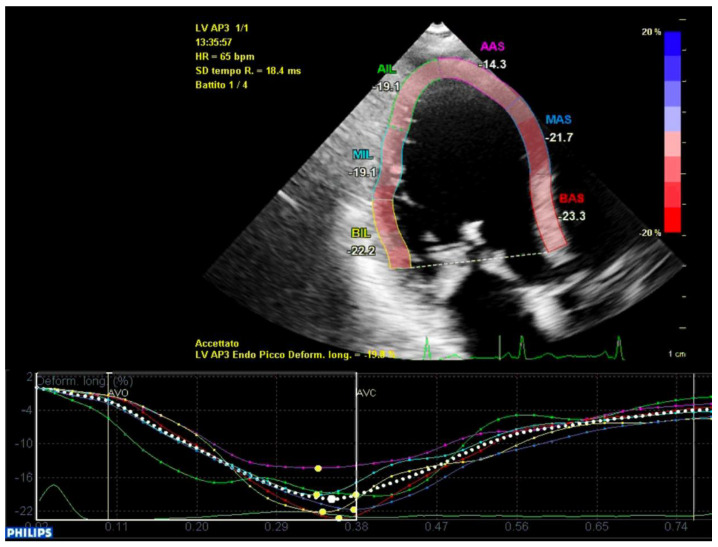
Global longitudinal strain in a patient from the A-MVP group. Mechanical dispersion is lower as peak systolic shortening times present lower variability.

In conclusion, the variables that were identified as the predictors of major arrhythmic events with the highest AUC (>0.80) within our high-risk population of arrhythmic MVP patients were the AML length (measured as shown in Figure 7), the S3 of the inferolateral basal segment and the mechanical dispersion of the basal and mid-ventricular segments calculated with speckle tracking analysis (Figure 8 and Figure 9).

The cut-off values matching the best sensitivity and specificity for each variable are shown in Table 5.

Interestingly, despite being the most well-known one, the inferolateral basal segment S3 velocity was proven to be a poor predictor of major arrhythmic events in terms of specificity compared to AML length and to mechanical dispersion of the basal and mid-ventricular segments measured with strain analysis. 

## 4. Discussion

The main goal of our study was to identify the echocardiographic parameters that are able to predict major arrhythmic events within MVP population at high risk of malignant arrhythmias. These patients presented several risk factors: they were predominantly women (19/22), with a bMVP mainly with redundant leaflets, and they all presented with a high ventricular ectopic burden during the ECG monitoring. In the recent past, several research groups have focused their efforts on identifying the predictors of arrhythmias without differentiating between major arrhythmic events and minor arrhythmias. However, most arrhythmic MVP patients still do not experience malignant events throughout their entire lives. In accordance with the risk factors described by Kukavica et al. [18], we focused on the anatomical features and on the contraction abnormalities that could be related to a higher risk of malignant events. We have hypothesized that the anatomical features that could explain a greater traction on the papillary muscles were a greater leaflets’ length or thickness and larger MVA diameters, area, or perimeter. The difference between the two groups in AML length—along with a larger area of the MVA in the ICD group—seems to confirm this hypothesis, as a longer leaflet exerts more intense traction towards the atrium during the end-systole period, favoring the formation of fibrosis and triggering VAs. At the same time, a larger MVA area leads to greater systolic forces on the valve, which cause greater leaflet displacement towards the atrium, and therefore, greater traction on the papillary muscles. Additionally, according to this hypothesis, a smaller value of the MV AP diameter/AML length ratio would indicate that the anterior leaflet might have a more curved leaflet shape, causing more abrupt tugging of the papillary muscle. Interestingly, the two groups did not differ neither in terms of MAD prevalence, nor in entity of MAD. A possible explanation may be found in the possible confusion between MAD and pseudo-MAD (with the over-diagnosis of MAD) [19].

The functional measurements included the research of a different mechanical dispersion (MD) between the two groups. We applied a protocol that has previously been described in different arrhythmic pathologies [17]. This protocol has the merit of matching an ECG marker (which reflects electrical activation) with an echocardiographic marker (which reflects the mechanical contraction of the segment analyzed), thus providing a multi-modality comprehensive index. Unfortunately, the TDI analyses did not identify any MD difference, probably because the population studied was already at high risk for arrhythmias. Additionally, a possible explanation for this is the fact that the TDI analysis focused on the mechanical contraction of the basal segments alone, which are distant from papillary muscle insertions, and thus are probably less affected by the anomalous stretching forces. These forces likely have greater effect on the middle segments, which play an important role in the mechanical triggering of arrhythmias.

In a recent publication by Ermakov et al. [20], speckle tracking was used to calculate the MD. Patients with arrhythmic MVP showed a significantly higher MD compared to patients with non-arrhythmic MVP, but the arrhythmic MVP group included patients with a fairly variable degree of arrhythmic burden. However, the analysis of MD within the arrhythmic MVP did not highlight a greater MD in patients with major arrhythmias. Our sub-analyses focused on the basal and mid ventricular segments were driven by the evidence that most of the arrhythmic substrate is located in the area between the papillary muscles’ insertion on the left ventricular free wall (the mid segments of the ventricle) and the area adjacent to the insertion of the leaflets (the basal segments). In fact, both of the areas are heavily stretched in the end-systole phase, respectively by the tugging of the papillary muscles and by the upper displacement of the leaflets. In accordance with our hypothesis, this is the area where most of the fibrosis is found in patients with MVP undergoing CMR or in autopsies of patients with MVP-related SCD. Therefore, speckle tracking seems the perfect tool to detect both the intensity of longitudinal traction and the variability of peak contraction time in the LV. An increased MD reflects a greater variability in the peak contraction time of the segments that are being analyzed, suggesting a pro-arrhythmic substrate in the areas where MD is more evident. In this view, the apical segments, which are not involved in the mechanical triggering of arrhythmia, act as a confounding factor that masks the differences between these two groups. In fact, we carried out sub-analyses measuring the MD of the basal segments alone, the mid segments alone and the apical segments alone, showing that the apical segments were the ones with the least difference in the MD. The basal segments and middle segments had both a higher level of discrepancy of MD between the two groups, but none of them reached a significant threshold when they were analyzed alone, supporting the hypothesis that the combination of both of the segments’ dyssynchronous contraction may be involved in the pathogenesis of major arrhythmic events.

Interestingly, in our population both the ROC curves of AML length and basal/mid-ventricular segments MD presented a similar AUC to that of the well-established S3 measurement. However, both variables provided a cut-off value with analogous sensitivity but substantially better specificity in the prediction of major arrhythmic events. 

In conclusion, the results of this study indicate the presence of new valuable echocardiographic parameters with a good ability to identify the patients at very high risk of malignant events within a high-risk features population. Nonetheless, all of the predictors that were previously described in other studies, despite not reaching significancy in our population, play an important role in selecting patients at risk of arrhythmic events within the general population of MVP. Ideally, both of the previously described predictors and the ones presented in our study may be integrated in a comprehensive evaluation of patients with MVP, allowing us to perform a more accurate arrhythmic risk stratification.

Echocardiography plays a pivotal role in arrhythmic risk stratification, but its diagnostic value needs to be integrated with other diagnostic tools such as ECG Holter monitoring, basal ECG, Cardiac Magnetic Resonance [16] and other tools that are able to predict the entity of adrenergic activation in patients such as ambulatory blood pressure monitoring or those that were demonstrated to be linked with an arrhythmic burden by Askin et al. [21].

An integrated approach that combines several diagnostic tools may lead to a more accurate stratification of arrhythmic risk in these patients and may represent a future development of the present study.

### Limitations

Our study presents some limitations, and the most important one is the small sample size. As it is a fairly rare condition, this is a limitation that is common in most of the studies investigating aMVP. A greater sample size would have allowed us to perform more robust statistical analyses. Additionally, another confounding factor is the fact that two patients in the aMVP group had undergone transcatheter ablation, which is likely to have a role in the reduction of major arrhythmic events.

## 5. Conclusions

MVP is a very common pathology that affects around 3% of the general population, and a small subset of these patients is at risk for malignant arrhythmic events. Despite the efforts that have been made in the recent years, a full comprehension of the mechanisms underlying MVP-related SCD is still lacking. Our study, which was conducted on a selected population of patients with high-risk features, identified five variables that were associated with increased arrhythmic risk: the MV annulus AP diameter/AML length ratio, the MV annulus indexed area, the inferolateral basal S3 velocity, the AML length and the basal/mid-ventricular segments mechanical dispersion calculated with speckle tracking analysis. These anatomical and functional features seem to be related, thus supporting the hypothesis of a mechanical stretch-induced origin of arrhythmic events. In fact, these findings suggest that VAs may be triggered by the abrupt tugging of the papillary muscle on the inferolateral wall. The last three parameters were the ones with the best AUCs in each respective ROC curves, and the last two parameters, in particular, provided cut-off values with the best sensitivity and specificity. These findings suggest that, among the population suffering from arrhythmic MVPs, echocardiography may identify patients at higher risk of major arrhythmic events. Further studies are needed to advance our understanding of the mechanisms of MVP-related VAs and SCD and to investigate additional risk factors.

## Figures and Tables

**Table 1:** Baseline clinical and echocardiographic variables analyzed in ICD-MVP group and A-MVP group.

Variables	ICD-MVP (6) Mean ± SD	A-MVP (16) Mean ± SD	*p*-Value
Female Sex (%)	83%	87%	0.83
Age (years)	43 ± 15	44 ± 14	0.83
BSA (m^2^)	1.6 ± 0.1	1.7 ± 0.1	0.44
LVED Volume (mL)	91 ± 17	88 ± 16	0.70
LVED Ind. Volume (mL/m^2^)	55 ± 7	52 ± 10	0.34
LVED Diameter (mm)	50 ± 2.5	49.5 ± 4.9	0.92
LVED ind. Diameter (mm/m^2^)	30 ± 2.6	29 ± 2.9	0.35
LV EF (%)	58 ± 3	59 ± 6	0.88
LV GLS (%)	18.1 ± 3.2	17.9 ± 2.5	0.85
LA ind. Volume (mL/mq)	44 ± 7	42 ± 17	0.69
Myxomatous Leaflets (%)	83%	81%	0.92

BSA: body surface area; LVED: left ventricular end-diastolic; LV: left ventricle; LA: left atrium; EF ejection fraction; GLS: global longitudinal strain.

**Table 2:** Anatomical echocardiographic parameters compared in ICD-MVP group and A-MVP group.

Variables	ICD-MVP Mean ± SD	A-MVP Mean ± SD	*p*-Value
AML Length (mm)	27.4 ±4.5	22.6 ± 2.9	0.01
AML Thickness (mm)	5.2 ± 0.3	5.0 ± 0.5	0.30
AP Diameter/AML Length Ratio	1.29 ± 0.3	1.47 ± 0.2	0.04
PML Length (mm)	16.3 ± 3.5	16.1 ± 3.1	0.86
PML Thickness (mm)	5.5 ± 0.8	5.0 ± 1.1	0.20
3D—AP ED Diameter (mm)	35.1 ± 1.2	31.5 ± 3.9	0.08
2D—AP ED Diameter (mm)	35.6 ± 2.2	32.9 ± 3.6	0.18
2D—AP ES Diameter (mm)	38.0 ± 1.9	35.0 ± 4.4	0.12
3D—IC ED Diameter (mm)	40.1 ± 1.9	37.9 ± 3.5	0.18
2D—IC ED Diameter (mm)	39.7 ± 2.5	37.2 ± 3.1	0.10
2D—IC ES Diameter (mm)	43.3 ± 3.0	39.8 ± 3.1	0.11
MVA ind. ED Circumference (mm/m^2^)	7.6 ± 1.2	6.7 ± 0.8	0.07
MVA ind. ED Area (cm^2^/m^2^)	7.0 ± 1.3	5.7 ± 1.3	0.02
MAD Prevalence	67%	56%	0.52
MAD Entity (mm)	10.2 ± 2.7	8.3 ± 3.7	0.19
Maximum Height Prolapse (mm)	5.4 ± 0.3	5.1 ± 0.5	0.74

AML: anterior mitral leaflet; AP: antero-posterior; PML: posterior mitral leaflet; 3D: three-dimensional; 2D: bi-dimensional; IC: inter-commissural; ED: end-diastolic; MVA: mitral valve annulus; MAD: mitral-annular-disjunction.

**Table 3:** Functional parameters comparison between ICD-MVP group and A-MVP group.

Variables	ICD-MVP Mean ± SD	A-MVP Mean ± SD	*p*-Value
Anterior S3 (cm/s)	18.1 ± 9.5	11.7 ± 6.9	0.07
Inferior S3 (cm/s)	21.5 ± 9.9	14.8 ± 9.9	0.20
Anterolateral S3 (cm/s)	21.5 ± 9.9	14.8 ± 9.9	0.07
Inferolateral S3 (cm/s)	28.2 ± 11.7	16.5 ±11.1	0.02
Anterior Septum S3 (cm/s)	9.4 ± 4.2	6.9 ± 2.5	0.12
Inferior Septum S3 (cm/s)	12.7 ± 4.7	9.4 ± 4.0	0.13
Q to S2 onset time (ms)	86.9 ± 9.7	76.9 ± 13.7	0.34
Q to S3 peak time (ms)	269.5 ± 28.6	266.4 ± 39.2	0.85
Q to end of S3 time (ms)	390.2 ± 18.8	389.7 ± 46.0	0.88
Q to S2 onset time SD (ms)	10.3 ± 4.8	11.8 ± 5.4	0.42
Q to S3 peak time SD (ms)	31.4 ± 30.5	32.0 ± 20.4	0.85
Q to end of S3 time SD (ms)	33.0 ± 20.2	21.1 ± 22.1	0.21
Grading of Mitral Regurgitation	3.0 ± 0.4	2.7 ± 0.9	0.18
Electro-Mechanical Window (ms)	9.2 ± 31.7	15.4 ± 39.1	0.13
Global MD—GLS (ms)	67.4 ± 23	70.0 ± 33	0.80
Basal MD—GLS (ms)	89.6 ± 15.9	63.3 ± 32.8	0.13
Mid MD—GLS (ms)	39.8 ± 30.8	39.2 ± 24.8	0.49
Apical MD—GLS (ms)	27.0 ± 20.6	47.2 ± 30.1	0.15
Basal/Mid-Ventricular Dispersion—GLS (ms)	116.7 ± 31.5	76.4 ± 48.4	0.03
Mid/apical Ventricular Dispersion—GLS (ms)	37.9 ±17.5	43.0 ± 21.4	0.54
Basal/apical Ventricular Dispersion—GLS (ms)	68.3 ± 11.9	61.7 ± 21.6	0.59
Paradoxical Systolic MV AP Diameter Expansion	1.07 ± 0.0	1.06 ± 0.1	0.86
Paradoxical Systolic MV IC Diameter Expansion	1.09 ± 0.1	1.07 ± 0.1	0.91

SD: standard deviation; MD: mechanical dispersion; GLS: global longitudinal strain; MV: mitral valve.

**Table 4:** Area under the curve (AUC) of ROC curves calculated for each significantly different variable between the two groups.

Variables	Area under the Curve (AUC)
AML Length	0.88
AP Diameter/AML Length Ratio	0.77
MVA indexed Area	0.79
Inferolateral Basal S3 velocity	0.81
MD of the basal and mid-ventricular segments	0.83

AML: anterior mitral leaflet; AP: antero-posterior; MVA: mitral valve annulus; MD: mechanical dispersion.

**Table 5:** Sensitivity and specificity of the best cut-off values identified for each predictor.

Variables	Cut-Off Value	Sensitivity	Specificity
Inferolateral basal segment S3	17 cm/s	83%	68%
AML Length	26.2 mm	83%	94%
Basal/Mid-Ventricular MD	122 ms	83%	88%

AML: anterior mitral leaflet; MD: mechanical dispersion.

## Data Availability

The study data will be made available upon request to the corresponding author.
